# In Vitro Evaluation of the Combination of *Melaleuca alternifolia* (Tea Tree) Oil and Dimethyl Sulfoxide (DMSO) against Trophozoites and Cysts of *Acanthamoeba* Strains. Oxygen Consumption Rate (OCR) Assay as a Method for Drug Screening

**DOI:** 10.3390/pathogens10040491

**Published:** 2021-04-19

**Authors:** Tania Martín-Pérez, Irene Heredero-Bermejo, Cristina Verdú-Expósito, Jorge Pérez-Serrano

**Affiliations:** Department of Biomedicine and Biotechnology, Faculty of Pharmacy, University of Alcalá, Alcalá de Henares, 28805 Madrid, Spain; irene.heredero@uah.es (I.H.-B.); cristina.verdu@uah.es (C.V.-E.); jorge.perez@uah.es (J.P.-S.)

**Keywords:** *Acanthamoeba* spp., genotype T3, genotype T4, cysts, dimethyl sulfoxide, tea tree oil, cytotoxicity, drug screening

## Abstract

Ameobae belonging to the genus *Acanthamoeba* are responsible for the human diseases Acanthamoeba keratitis (AK) and granulomatous amoebic encephalitis (GAE). The treatment of these illnesses is hampered by the existence of a resistance stage (cysts). In an attempt to add new agents that are effective against trophozoites and cysts, tea tree oil (TTO) and dimethyl sulfoxide (DMSO), separately and in combination, were tested In Vitro against two *Acanthamoeba* isolates, T3 and T4 genotypes. The oxygen consumption rate (OCR) assay was used as a drug screening method, which is to some extent useful in amoebicide drug screening; however, evaluation of lethal effects may be misleading when testing products that promote encystment. Trophozoite viability analysis showed that the effectiveness of the combination of both compounds is higher than when either compound is used alone. Therefore, the TTO alone or TTO + DMSO in combination were an amoebicide, but most of the amoebicidal activity in the combination’s treatments seemed to be caused mainly by the TTO effect. In fact, DMSO alone seems to be a non-amoebicide, triggering encystment. Regarding cytotoxicity, these compounds showed toxicity in human corneal epithelial cells (HCEpiC), even at low concentrations when tested in combination. In conclusion, the use of TTO and DMSO, in combination or alone, cannot be recommended as an alternative for AK treatment until more cytotoxicity and cyst adhesion tests are performed.

## 1. Introduction

The genus *Acanthamoeba* belongs to the group referred to as free-living amoebae (FLA) and also includes other important genera, such as *Balamuthia* and *Naegleria*. These organisms are environmentally widespread [1]. Some *Acanthamoeba* sp. isolates are opportunistic pathogens, occasionally causing *Acanthamoeba* keratitis (AK), a disease characterized by corneal inflammation, and granulomatous amoebic encephalitis (GAE), an infection of the central nervous system. The incidence of AK is increasing due to the growing numbers of contact lens wearers and the improvements in diagnostic methods [2]. In contrast to GAE, AK occurs in immunocompetent individuals [3]. The treatment of these infections is still problematic because FLA are more resistant when they are in the cyst stage. Trophozoites undergoing changes in temperature, lack of nutrients, adverse pH or chemical attack may quickly start to develop a cystic cover forming the typical double walled cyst [4,5]. Cysts are difficult to eradicate with disinfectants or drugs and it is regularly observed that protozoa remain alive after treatment of infected patients. Indeed, if not all trophozoites and cysts are removed, the infection remains active. 

While many compounds have been tested for treating the corneal surface and there are cleansing solutions for contact lenses, the treatment of AK remains elusive [6,7,8]. Products employed for local treatment of keratitis are preferentially biguanides (namely polyhexamethylene biguanide (PHMB) and chlorhexidine) or diamidines (propamidine and hexamidine) [9]. The mode of action of these drugs is by damaging the cellular membranes [10]. Unfortunately, many commercial solutions for lens hygiene containing PHMB were ineffective against *Acanthamoeba* contamination in contact lenses, as demonstrated by a study conducted in Korea [11]. Similar findings have been observed for many multipurpose cleansing solutions in other studies [12,13,14,15,16].

Indeed, the panorama is not totally negative, as some hygienic solutions have been shown to be effective in other studies [17,18]. There is a wide consensus that the development of new antiseptic products is essential to increase protection for contact lens users as well as new drugs to avoid treatment failure. Additionally, effective treatment in patients has been achieved [19,20]. 

To broaden the field of new and effective treatments, a variety of different chemicals and natural products have been tested. The most recent advances in this field indicate that cationic dendrimers [21,22] or plant extracts such as tea tree oil (TTO) [23,24] might be useful for prophylaxis of AK. The use of dimethyl sulfoxide (DMSO) as a disinfectant was proposed by Siddiqui et al. [7]. In these studies, a T4 *Acanthamoeba* strain was used. It is well known that non-T4 genotypes have a worse response to medical therapy [25]. With this in mind, the amoebicidal effect of TTO and DMSO, tested individually, was evaluated in a T3 and T4 genotype strain. Additionally, the effect of TTO and DMSO in combination was studied in T3 and T4 *Acanthamoeba* strains, as well as their cytotoxic effect in human corneal epithelial cells (HCEpiC). Moreover, the oxygen consumption rate (OCR) assay was evaluated as a method for drug screening in order to know if manual counting could be replaced. This assay was previously tested by Heredero-Bermejo et al., [26] with a good correlation with manual counting.

## 2. Results

### 2.1. Trophocidal Properties of Tea Tree Oil (TTO) and Dimethyl Sulfoxide (DMSO)

**Figure 1:**
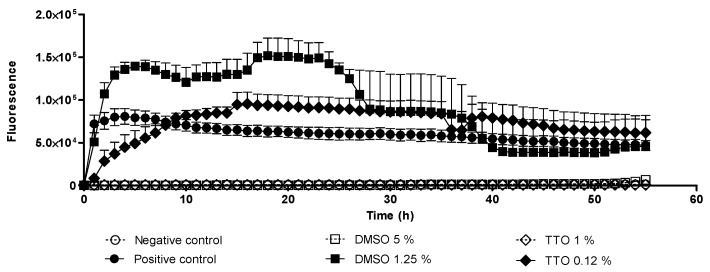
Respiration plot of *A. griffini* MYP2004 treated with tea tree oil (TTO) and dimethyl sulfoxide (DMSO).

**Figure 2:**
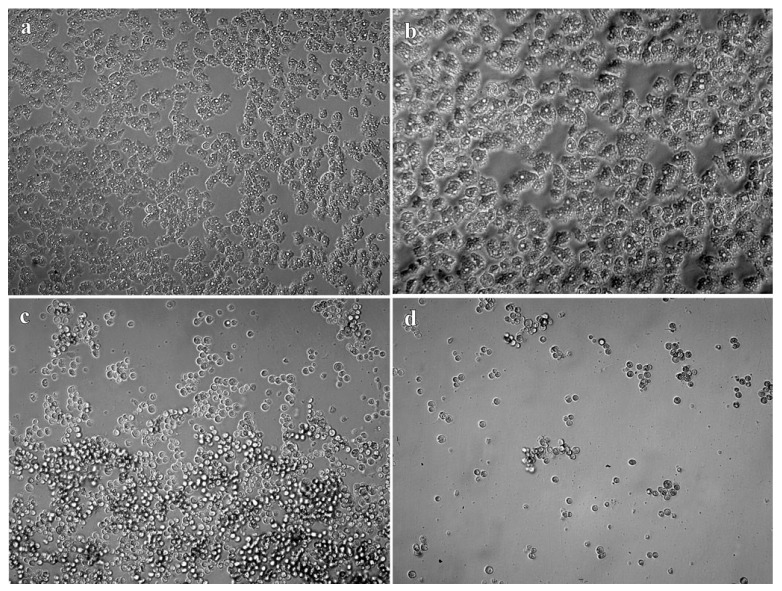
DMSO treatment in trophozoites of *A. griffini* MYP2004. Photographs obtained under the light microscope. (**a**) Trophozoites observed in the control. (**b**) Trophozoites treated with 0.625% DMSO. (**c**) Cysts observed at a concentration of 1.25% DMSO. (**d**) Cysts observed at a concentration of 2.5% DMSO.

**Figure 3:**
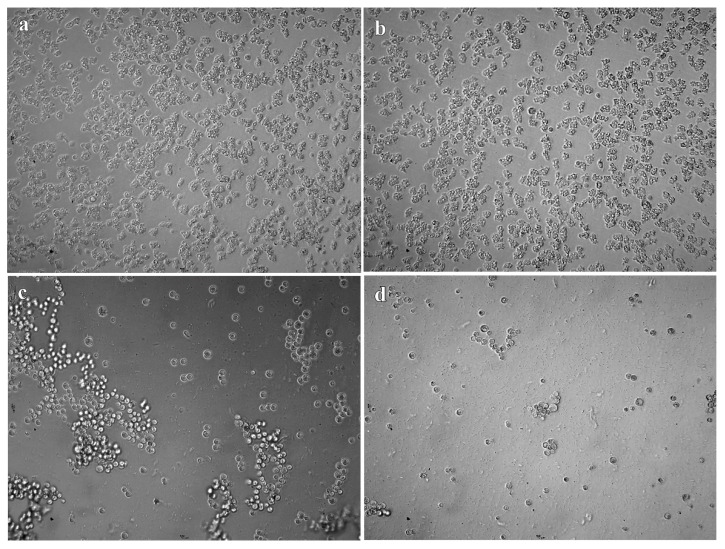
DMSO treatment in trophozoites of *A. polyphaga* 2961. Photographs obtained under the light microscope. (**a**) Trophozoites observed in the control. (**b**) Trophozoites treated with 0.625% DMSO. (**c**) Cysts observed at a concentration of 1.25% DMSO. (**d**) Cysts observed at a concentration of 2.5% DMSO

According to OCR results, the fluorescence levels (Figure 1) obtained in the positive control wells, and in the treated with 0.12% of TTO and 1.25% DMSO were higher than the values obtained at other concentrations. These high fluorescence values indicated a higher rate of oxygen consumed which implies a greater number of viable trophozoites. No fluorescence emission was observed in the negative control wells as well as in the wells treated with 1% TTO and 5% DMSO, which indicates that these concentrations were amoebicidal. Whereas the putative amoebicidal effect was observed under light microscope, the higher DMSO concentrations tested did not kill trophozoites, they merely promoted encystment. The cysts observed under microscope (Figure 2 and Figure 3) revels cysts with a single layer instead two layers, these types of cyst are called pseudo-cysts [27]. However, in the wells treated with 1% TTO, no viable cysts or trophozoites were observed.

**Figure 4:**
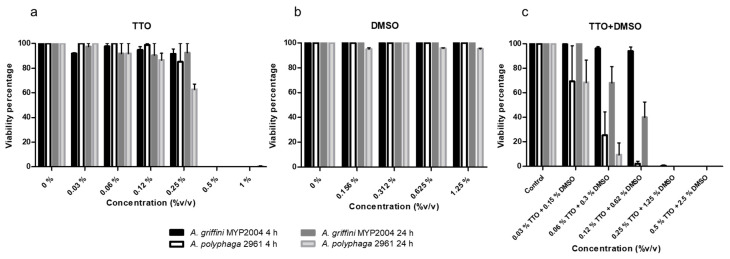
Viability percentages of *A. griffini* MYP2004 and *A. polyphaga* 2961 after 4-h and 24-h treatment. (**a**) Treated with TTO. (**b**) Treated with DMSO. (**c**) Treated with TTO + DMSO.

The amoebicidal activity of TTO and DMSO tested in combination and separately was evaluated also by manual counting (Figure 4). TTO was able to kill all trophozoites at a concentration of 0.5% after 4- and 24-h treatments (Figure 4a). In contrast, DMSO has no amoebicidal effect on *Acanthamoeba* trophozoites at 4 and 24 h (Figure 4b). The combination of both compounds is more effective than when they are applied separately. The effect of TTO in combination with DMSO causes lysis of trophozoites at DMSO concentrations at which encystment is favored (0.25% TTO + 1.25% DMSO). The efficacy of the drug combination is not dose-dependent, since a reduction in viability percentage is not observed until the concentration 0.12% TTO + 0.625% DMSO (Figure 4c). The differences observed between the treatment at 4 and 24 h are not statistically significant, even though a greater reduction in trophozoites viability treated at 24 h is observed. Differences are also observed between the genotype T3 (*A. griffini* MYP2004) and genotype T4 (*A. polyphaga* 2961), being more resistant the genotype T3, but these differences are not statistically significant (Table 1).

As a greater effectiveness was observed when both compounds were applied together, the fractional inhibitory concentration index (FICI) was calculated (Table 2). The FICI values indicates that there is no synergy effect.

### 2.2. In Vitro Cysticidal Assays

All concentrations tested in amoebicidal assays were also tested against cysts. Unfortunately, TTO and DMSO tested in combination and separately were not effective against cysts. After 3 days of incubation, trophozoites were observed at all concentrations. Hence, a complete reversion of cysts to trophozoites was not avoided even at higher concentrations.

### 2.3. Cytotoxicity Test on Human Corneal Epithelial Cells

**Figure 5:**
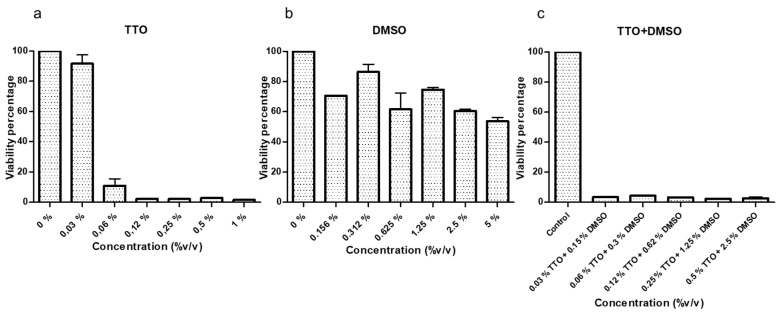
Cytotoxic test in human corneal epithelial cell (HCEpiC) line. (**a**) Treated with TTO. (**b**) Treated with DMSO. (**c**) Treated with TTO + DMSO.

A cytotoxicity test in HCEpiC showed that all concentrations of TTO were of high toxicity, except the concentration of 0.03% (Figure 5a). The DMSO showed low and moderate toxicity in the concentrations that triggers the encystment (Figure 5b). Moreover, a low viability percentage was observed when cells were treated with TTO and DMSO in combination even at low concentration, so it was classified as high toxicity (Figure 5c). Hence, the concentrations that were able to kill 100% of trophozoites also killed 100% of HCEpiC. 

## 3. Discussion

The lack of a standard treatment for AK and GAE has led to a search for alternative treatment methods. In recent years, numerous compounds with trophocidal activity have been reported, but many of these are not effective against *Acanthamoeba* cysts [24]. Among these compounds, TTO and DMSO appear promising, which is why this study was carried out.

The antimicrobial activity and cytotoxicity of TTO has been widely studied [28,29], and there is no doubt about the bactericidal and bacteriostatic effect against various bacterial species, as well as its antifungal capacity [28]. Likewise, a notable acaricidal effect was reported [30], the simple exposure of TTO vapors has an effect on mites [31]. Moreover, anti-*Acanthamoeba* activity was reported [23,32]. In our study, an anti-*Acanthamoeba* activity was also observed at 0.5% concentration. In the study of Hadaś et al. [23], TTO showed cysticidal effect unlike what we observed in our work. The treatment of Hadaś et al. [23] consisted of adding TTO for 5 days, while in our study TTO was only added once. Therefore, this could be the reason why cysticidal activity was not observed. Moreover, it is not known which components of TTO are responsible for this activity and if they act solely or synergistically [32]. Regarding the toxicity of TTO, in our study TTO solution was cytotoxic in HCEpiC cells at a very low concentration (0.06%). Previous cytotoxic studies have demonstrated that the ingestion of high amounts of this compound is toxic and can cause allergic reactions in topical use [28]. Furthermore, cytotoxicity was studied in human cell lines such as monocytes and neutrophils in which TTO was cytotoxic at concentrations of 0.016% (*v*/*v*) which are even lower than the concentrations tested in our study [33]. However, the relationship between In Vitro and in vivo cytotoxicity is unknown. On the other hand, TTO is commonly used topically, and the application in any other way, such as eye drops in an in vivo model for treating AK, must be carefully studied before being applied since there are not enough studies in this regard [29]. Based on our findings, suggesting the use of TTO as a possible candidate for treating AK is risky, as it showed cytotoxicity in an In Vitro test and its in vivo cytotoxicity is not really known. Thus, more In Vitro and in vivo cytotoxicity tests should be carried out. In future studies, lower TTO concentrations will be applied repeatedly at different times during a few days in order to know if these treatment conditions are effective and non-toxic.

Regarding to DMSO, it is an organic solvent which has a wide range of primary pharmacological actions such as membrane penetration, membrane transport, anti-inflammation, nerve blockade, bacteriostasis, muscle relaxation, among others [34]. The DMSO was proposed by Siddiqui et al. [7] to be included as a component of lens cleaning solutions since it triggers encystment and, some studies have confirmed that cysts are unable to adhere to corneal epithelial cells [35]. In this study, it was observed that the concentration that favors encystment is lower than that indicated by Siddiqui et al., [7]. Furthermore, it was observed that DMSO is not cytotoxic in human corneal epithelial cells at these concentrations. However, we consider that DMSO should not be included in contact lens solutions because other studies found that cysts are able to adhere to contact lens [36,37]. Moreover, Dudley et al., [35] indicated that “Adhesion assays revealed that *Acanthamoeba* cysts belonging to T1, T2, T3, T4, and T7 genotypes exhibited no and/or minimal binding (<5%) to the host cells”. Hence, although the adhesion of the cysts is minimal, there is a potential risk that *Acanthamoeba* cysts would excyst and could invade the corneal epithelium, developing AK infection. Moreover, to support this affirmation, an outbreak in the USA was related to a contact lens solution that contained propylene glycol, a compound that produced *Acanthamoeba* trophozoites encystment [38].

As far as we know, this is the first study in which TTO and DMSO were tested in combination. The combination therapy of both compounds revealed a better trophocidal activity than when each compound was applied separately. Nonetheless, the FICI index indicated that there was not a synergic effect at any of these effective combinations. In addition, it is important to remark that DMSO did not promote the encystment of the trophozoites at these combinations, and the combinations did not have cysticidal effects.

Finally, the OCR plates were previously proposed as a drug screening method being to some extent useful in amoebicide drug screening; however, evaluation of lethal effects may be misleading when tested products promote encystment. Therefore, parallel microscopic observations must always accompany any study based on respiration analysis. 

## 4. Materials and Methods

### 4.1. Acanthamoeba Strains

The strains used were *A. griffini* MYP2004-T3, isolated from a contact lens from a patient with AK in Spain [39], and *A. polyphaga* 2961-T4, a clinical isolate kindly supplied by Dr. E. Hadaś (Poznan University of Medical Sciences, Poland). *A. griffini* was cultured axenically in CERVA medium (20 g L^−1^ bactocasitone, 10% foetal bovine serum (FBS), 0.1 g L^−1^ streptomycin and 0.06 g L^−1^ penicillin [39] and maintained at 37 °C, while *A. polyphaga* was cultured axenically in Peptone-Yeast-Glucose (PYG) medium (10 g L^−1^ east extract, 5 g L^−1^ D-glucose, 10 g L^−1^ protease peptone, 5 g L^−1^ NaCl, 3.57 mg L^−1^ Na_2_HPO_4_, 3.45 mg L^−1^ KH_2_PO_4_, 0.1 g L^−1^ streptomycin and 0.06 g L^−1^ penicillin) supplement with 2% bactocasitone (PYG-B) [40] and maintained at 32 °C.

Cysts were obtained by culturing 7- to 10-day trophozoites in Neff’s encystment medium [41].

### 4.2. In Vitro Amebicide Assays

The effect of TTO and DMSO was evaluated in combination and individually in two *Acanthamoeba* strains (*A. griffini* MYP2004 and *A. polyphaga* 2961). Firstly, two concentrations of TTO (0.12% and 1%) and DMSO (1.25% and 5%) alone were evaluated by oxygen consumption rate (OCR) assay in *A. griffini* MYP2004 because this genotype is more resistant than *A. polyphaga* 2961 and the aim was to determine the range of concentrations to be tested and not the efficacy of each compound. The OCR assay was previously described by Heredero-Bermejo et al. and Martín-Pérez et al. [23,42]. Briefly, 50 µL of CERVA medium containing 10^5^ amoebae from log phase cultures (grown for 48–72 h) was added to 50 µL of drug solution and loaded into the wells of 96-well microplates (Oxoprobics Biosciences, Madrid, Spain). Then, wells were overlaid with 100 µL of mineral oil to avoid oxygen replenishment from air. Unless otherwise stated, each concentration of TTO and DMSO was assayed in triplicate and repeated in at least two independent experiments. The microplate was placed in a fluorescence reader (VICTOR^®^ PerkinElmer, Waltham, MA, USA), which was programmed to obtain two readings per well and per hour (for a period of 55 h) at 37 °C. Time-resolved fluorescence (excitation at 340 nm/emission at 642 nm) was measured at delay times of 30 and 70 microseconds. 

Then, the amoebicidal activity of DMSO and TTO was assessed by direct counting, using the 0.2% Congo red exclusion assay [41]. It was assayed in sterile 24-well microplates. Diverse drug concentrations (% *v*/*v*) were prepared via serial dilution in culture media (CERVA or PYG + B) that in the case of the TTO concentrations were 1–0.03% and in the case of the DMSO 5–0.156%. In addition, combinations of TTO and DMSO were analyzed. The concentrations were serial diluted ranging from 0.5% TTO + 2.5% DMSO to 0.03% TTO + 0.15% DMSO.

Amoeba from log-phase cultures (1·10^5^ trophozoites/well) were inoculated into the microplates. The trophozoites were resuspended in the culture medium. Drugs assays contained 300 µL of trophozoites solution and 300 µL of the drug solution per well. There was a control for growth (positive control), including only medium and amoebae. Finally, the negative control was composed of medium but contained no amoebae. Each drug concentration and controls were done in triplicate and each experiment was undertaken at least twice in independent experiments. The plates were sealed with Parafilm^®^ and incubated at 32 or 37 °C (depending on the *Acanthamoeba* strain) during 4 and 24 h. Samples were placed in a Fuchs–Rosenthal manual counting chamber and trophozoites counted using an optical microscope (Carl Zeiss). The minimum trophozoite amoebicidal concentration (MTAC) was defined as the lowest concentration of test solution that produced a complete destruction of trophozoites [43].

### 4.3. In Vitro Cysticidal Assays

The experiments to test drugs on cysts were performed in sterile 96-well microtiter plates. Cysts were resuspended in 1× phosphate buffered saline (PBS) at a concentration of 10^5^ cysts/mL and 100 μL of the calibrated cyst suspension were added to each well (10,000 cysts per well). Control wells received 100 μL of 1× PBS (vehicle) instead of drug solutions. Then, the plates were sealed with Parafilm^®^ and incubated for 24 h. Assays were performed in triplicate and were repeated at least twice.

The viability of treated cysts was studied by assessing excystment. Wells were washed twice with 1× PBS to eliminate residual drugs. Then, 200 μL of fresh CERVA or PYG + B medium were added to each well. Plates were incubated and observed microscopically daily for 21 days to calculate the minimum cysticidal concentration (MCC), defined as the lowest concentration of test solution at which cyst excystment and trophozoite growth are completely inhibited [43].

### 4.4. Cytotoxicity Test on Human Corneal Epithelial Cells

The non-cytotoxic dose of the compounds was determined using human corneal epithelial cells (HCEpiC) (Innoprot, ref. P10871). Experiments were performed in 24-well microplates. The HCEpiC were grown in corneal epithelial cell medium (CEpiCM) (Innoprot, ref. P60131) supplemented with 1% of corneal epithelial cell growth supplement (CEpiCGS), 2% of fetal bovine serum (FBS) and 1% antibiotic mix: 10,000 U penicillin, 10 mg streptomycin and 25 µg amphotericin B per milliliter. HCEpiC were seeded at a density of 2 × 10^4^ cells/well in 500 µL medium. 

After incubating the plates for 3 days at 37 °C in a 5% CO_2_ atmosphere to form a confluent cell monolayer, the medium was replaced by 250 µL of fresh medium plus 250 µL of the compounds dissolved in CEpiCM. Controls wells received 250 µL of media instead of drugs. After 24 h of incubation, the cytotoxicity was evaluated using the microculture tetrazolium assay (MTT), adding 50 µL of MTT (5 mg/mL) to each well. After 3 h of incubation at 37 °C, the content of each well was discarded and 500 µL of dimethyl sulfoxide (DMSO) was added to dissolve formazan crystals and the absorbance of the samples recorded in a microplate absorbance reader at 570–630 nm (BioTek Instruments Inc. Model: ELX 800, Winooski, VT, USA).

It was considered that viability values between 0–10% were non-toxic, 10–25% low toxicity, 25–40% moderate toxicity, and higher than 40% was considered high toxicity.

### 4.5. Calculation of In Vitro Fractional Inhibitory Concentration Index (FICI)

The data obtained from the amoebicidal studies were interpreted by calculating the fractional inhibitory concentration index as follows (FICI): FICI = (MTAC drug A combination/MTAC drug A alone) + (MTAC drug B combination/MTAC drug B alone). The index was interpreted as follows: FICI ≤ 0.5, synergism; FICI > 0.5 to ≤2.0, indifference; FICI of >2.0, antagonism [44].

### 4.6. Statistical Analysis

All experiments were performed in triplicate. Results are given as means ± SD of values obtained from two independent experiments. The significance of differences to control was determined using a two-way analysis of variance (ANOVA) test for independent samples and two-tailed Student’s *t*-test. Statistical significance was defined as *p* < 0.05. Statistical analysis was carried out using GraphPad Prism 5^®^ (GraphPad Spftware, San Diedo, CA, USA).

## 5. Conclusions

Even though TTO has an amoebicidal activity (alone and in combination with DMSO), it also showed a cytotoxicity effect on human corneal epithelial cells. Therefore, its use might not be suitable as an alternative for treating AK until more information about its cytotoxicity is known. DMSO triggers trophozoites encystment and, although adhesion of the cysts is minimal, there is a potential risk that *Acanthamoeba* cysts would excyst and invade the corneal epithelium, developing AK infection. Accordingly, the use of DMSO is not recommended to treat AK. Regarding the OCR assay, the use of metabolic methods to assess the viability of trophozoites should always be accompanied by microscopic observations performing a dye exclusion test.

## Figures and Tables

**Table 1:** The minimum trophozoite amoebicidal concentration (MTAC) of TTO, DMSO and TTO + DMSO in trophozoites of *A. griffini* MYP2004 and *A. polyphaga* 2961 after 24 h of treatment. The minimum cystidal concentration (MCC) of TTO, DMSO and TTO + DMSO after 24 h of treatment. Data shown % *v*/*v*.

	*A. griffini* MYP2004		*A. polyphaga* 2961	
	MTAC	MCC	MTAC	MCC
TTO	0.5	>1	0.5	>1
DMSO	>1.25	>1.25	>1.25	>1.25
TTO+DMSO	0.25 + 1.25	>0.5 + 2.5	0.12 + 0.625	>0.5 + 2.5

**Table 2:** Fractional inhibitory concentration index (FICI) of the combination therapy of TTO and DMSO against *A. griffini* MYP2004 and *A. polyphaga* 2961.

***A. griffini* MYP2004 (Genotype T3) MTAC (% *v/v*)**				**FICI**	**Interpretation**
**TTO (Alone)**	**DMSO (Alone)**	**TTO** **(Combination)**	**DMSO** **(Combination)**		
0.5	1.25	0.25	1.25	1.5	Indifference
***A. polyphaga* 2961 (Genotype T4) MTAC (% *v/v*)**				**FICI**	**Interpretation**
**TTO (Alone)**	**DMSO (Alone)**	**TTO** **(Combination)**	**DMSO** **(Combination)**		
0.5	1.25	0.12	0.625	0.74	Indifference

## Data Availability

Not applicable.
